# Bayes’ theorem, the ROC diagram and reference values: Definition and use in clinical diagnosis

**DOI:** 10.11613/BM.2018.010101

**Published:** 2017-11-24

**Authors:** Anders Kallner

**Affiliations:** Department of clinical chemistry, Karolinska University Laboratory, Stockholm, Sweden

**Keywords:** likelihood ratio, cumulative data analysis, odds, sensitivity, specificity

## Abstract

Medicine is diagnosis, treatment and care. To diagnose is to consider the probability of the cause of discomfort experienced by the patient. The physician may face many options and all decisions are liable to uncertainty to some extent. The rational action is to perform selected tests and thereby increase the pre-test probability to reach a superior post-test probability of a particular option. To draw the right conclusions from a test, certain background information about the performance of the test is necessary. We set up a partially artificial dataset with measured results obtained from the laboratory information system and simulated diagnosis attached. The dataset is used to explore the use of contingency tables with a unique graphic design and software to establish and compare ROC graphs. The loss of information in the ROC curve is compensated by a cumulative data analysis (CDA) plot linked to a display of the efficiency and predictive values. A standard for the contingency table is suggested and the use of dynamic reference intervals discussed.

## Introduction

Bayes’ theorem pertains to calculating and describing the gain in probability of correct prediction of an event before and after performing a test designed to be specific for the event. “Event” shall be understood in the widest of possibilities *e.g.* rain or no rain, health or disease, true radar echo or false.

Prediction is the essence of diagnosis; a preliminary diagnosis can be confirmed or discarded with a defined uncertainty considering the test result and its potential. Application of Bayes’ principle and its use in laboratory medicine is intuitive, but data collection, their systematic use and understanding are not always that easy. Its introduction to laboratories should rightly be ascribed to Galen and Gambino (1976) and their seminal treatise “Beyond normality” (*1*).

History tells that receiver (or relative) operating (or operator) characteristics (ROC) plots were first used before and during the Second World War in order to optimize the signal to noise ratio in developing the radar technique, *i.e.* identifying true objects as different from false “blips”. Exactly how this was done is outside the scope of this presentation but most likely the intensity or frequency of a signal was quantified and compared to a threshold value defined to be fit for purpose. The use of ROC graphs was soon adopted in Signal Detection Theory but was not introduced in health sciences and related research fields until the early 1970s. ROC curves were much discussed during the 1990s (*2*, *3*). The ROC plot itself is a convenient tool to illustrate the ability of a test to enhance a pre-test probability, predict the post-test probability and illustrate the Bayes’ theorem in practical diagnostic applications. It is particularly useful in optimizing a threshold value between two conditions *e.g.* health and non-health, *i.e.* definition of reference values whereas the actual value of the cut-off cannot be read directly from the graph. The ROC technique is also used in comparing clinical usefulness of diagnostic procedures by calculating the area under the curve, which can be understood as an expression of correct ranking and ability to discriminate. In this report, we discuss and display the Bayes’ theorem and explore some features of the ROC by innovative graphs with reference to establishing reference values.

## Data collection and study design

**Figure 1:**
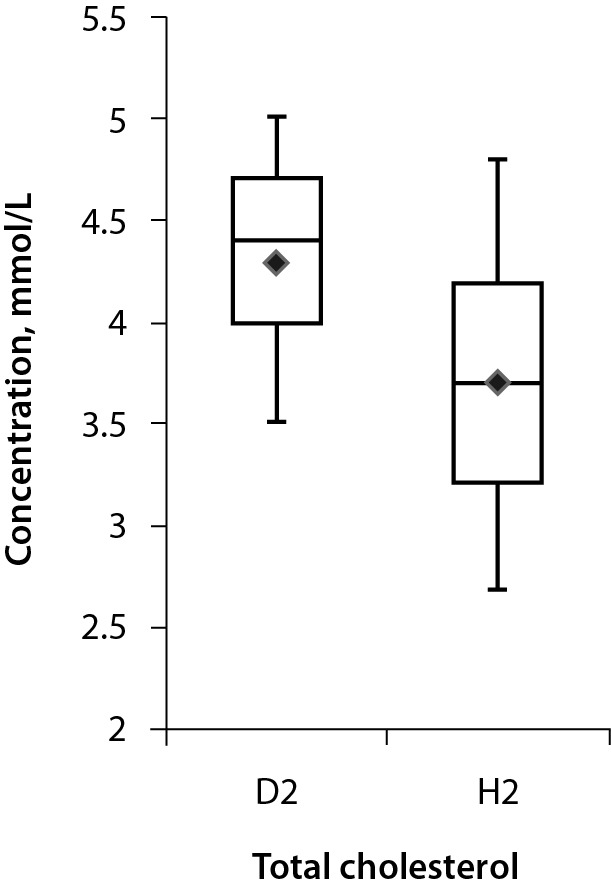
Box-plot of the test dataset after artificial grouping into diseased (D2) and healthy (H2) individuals. The median (horizontal lines across the box), arithmetic mean (diamond shapes), interquartile ranges (boxes) and minimum and maximum values (whiskers) of the groups are indicated.

Data required for defining and evaluating a diagnostic marker are the definitive numbers of diseased and not diseased individuals identified by an independent procedure, cross-referenced to the biomarker. To define a suitable cut-off, the reference population needs to be representative of the population and situation in which the marker is intended to be used, *e.g.* screening, emergency, children *etc.* For illustration, we created a partly artificial dataset based on a large number of consecutive, unidentified plasma total cholesterol values obtained during a limited time from the laboratory information system (LIS) (*4*). We selected all values between 2.8 and 5.0 mmol/L (in total about 10,000 results) as possibly hypercholesterolemic. Since the study design did not include a clinical diagnosis, the patients’ diagnoses were assigned by simulation. This was designed to ensure that the “diseased” were represented by higher values, *i.e.* the distribution of diseased was skewed to the right. Eventually, we randomly selected 2500 individuals classified as not diseased and 2500 as diseased from the initial cohort. This resulted in a prevalence of disease of 50%. The structure of the dataset is illustrated by the box-plot in Figure 1.

The obtained dataset was then entered into an Excel spreadsheet program which assigns the data to a 2x2 contingency table and allows the use of variable cut-offs. The program also presents a ROC-curve, based on the calculated sensitivity and specificity, a box-plot of the healthy and diseased groups, cumulative data analysis (CDA) and efficiency graphs, a comparison between two performance studies with a possibility to visually include four additional studies.

## The contingency table

**Figure 2:**
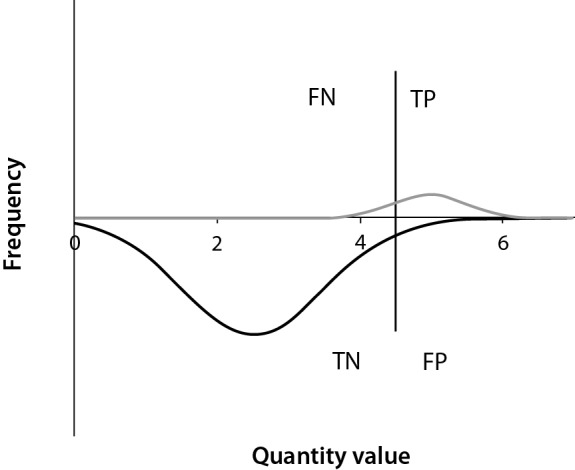
Graph designed to mimic a contingency table for different outcomes marked. The diseased population is above the X-axis and characterized by high results. The cut-off is marked as a vertical line. Results on the left would be called ”negative” and those on the right ”positive”. Thus, false negatives (FN) are found in the small area under the curve to the left of the cut-off line and above the X-axis. False positives (FP) are found above the curve but under the X-axis to the right of the cut-off. Think of moving the cut-off to a new position and note that both the FN and the FP are changed but in different directions. *Nota bene*, it is unlikely that the quantity values of the diseased group will be normally distributed.

The contingency table was organized with categories, *e.g.* the number of diseased in one row and the healthy (non-diseased) in the row below. The test results, *i.e.* the number of negative and positive results in relation to a particular quantity value, the cut-off, are given in columns (Table 1). The four outcomes are true results *i.e.* healthy and diseased correctly classified (true negative and true positive, respectively) and false results, healthy individuals classified as diseased (false positive *i.e.* Type I error) and diseased that are not identified (false negative *i.e.* Type II error). The data presented in Table 1 is also shown in corresponding graphical form in Figure 2.

**Table 1:** Model of a contingency (2x2) table

	**Negative test**	**Positive test**	
**Diseased**	FN	TP	Sensitivity, TP / D
**Healthy**	TN	FP	Specificity, TN / H
	PV(-), TN / NEG	PV(+), TP / POS	
FN – false negative. TP – true positive. FP – false positive. TN – true negative. FN, TP, FP and TN are expressed as numbers. PV(-) - negative predictive value. PV(+) - positive predictive value. NEG – total number of negatives, *i.e.* NEG = FN + TN. POS - total number of positives, *i.e.* POS = TP + FP. D - total number of diseased, *i.e.* D = FN + TP. H - total number of healthy, *i.e.* H = TN + FP. Total population sample N = FN + TN + TP + FP.			

## Calculated quantities

The contingency table summarizes the findings and offers keys to statistical and practical inference. The most important quantities, or indices or performance characteristics, are the “true positive rate” or “sensitivity” (TPR) and the “true negative rate” or “specificity” (TNR), both calculated along the rows as the ratio between the number of true observations and all healthy or diseased, respectively. From the columns the negative PV(-) and positive predictive value PV(+) are obtained (Table 1 and Appendix 1). The “efficiency” or "accuracy" (deprecated term) is the true result rate, *i.e.* the ratio between the total number of true results and all observations. The relative number of diseased in the reference population is the prevalence of disease in the population if the reference sample population is representative for that population.

**Figure 3:**
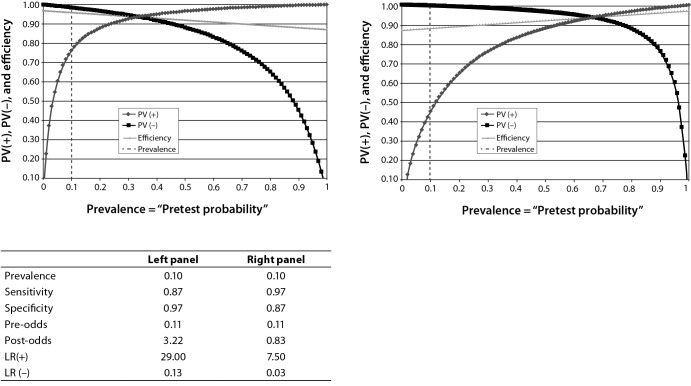
Outcome in terms of predictive values at a fixed prevalence of 10% and interchanging sensitivity and specificity values. Clearly, if the sensitivity is increased (right panel) the overall performance (efficiency) drops as the likelihood decreases. This favours the ruling out of not diseased, the FN drops. PV (+) – positive predictive value. PV (-) – negative predictive value. LR (+) – positive likelihood ratio. LR (-) - negative likelihood ratio. The vertical dashed line represents the prevalence of disease and increasing the prevalence will improve the performance.

The sensitivity and specificity are regarded as constants for a defined measuring system used for a clinical problem, *i.e.* the disease or condition in question. They are interrelated; if one is increased, the other will decrease (Figure 3), therefore, both cannot be increased (or decreased) simultaneously. Altering the cut-off value will change the relation between sensitivity and specificity but not the total number of diseased and not diseased, *i.e.* the number of observations in the rows. Therefore, the relation between the number of diseased and not diseased depends on the prevalence of disease.

The predictive values are closely related to the prevalence of disease and thus the relation between diseased and healthy individuals. Therefore, the numbers are only valid in a given cohort. They will change if the disease is redefined or the prevalence changes.

There are mathematical relations between the performance characteristics (Appendix 1) but they are not necessarily of practical value. Given the sensitivity, specificity and prevalence of disease, the complex pattern of predictive values can be illustrated as in Figure 3.

## Bayes’ theorem

The calculated quantities in a contingency table allow us to resolve the relation between the pre-test probability and the post-test probability. In the present context, Bayes’ theorem is used to estimate the gain in the probability of a correct prediction that is achieved by performing and inferring the results of an investigation. This requires the definition of factors known as positive and negative “likelihood ratio”. They are defined as the ratio of the true positive rates (TPR) to false positive rates (FPR), and false negative rates (FNR) and true negative rates (TNR), respectively. In laboratory medicine these terms are better known as the sensitivity (or TPR), (1 – specificity) (or FPR), (1 – sensitivity) (or FNR) and specificity (or TNR), respectively (see Appendix 1). Only the true rates (*i.e.* sensitivity and specificity) are directly calculated from the contingency table and listed as marginal quantities in a separate column.

The positive likelihood ratio (LR(+)) is the essence of Bayes’ theorem and states that the post-test probability equals the pre-test probability multiplied by the likelihood ratio. Commonly, only tests with a LR(+) of 3 or more are considered for a positive diagnosis. The calculations are inconvenient because they require that the probabilities are expressed as odds, whereas scientists and laboratorians are more familiar with probabilities expressed for instance as percentage (Appendix 1). A useful, ingenious nomogram is available as a calculator or diagram on many internet sites, designed by Fagan (*5*). Most published Fagan diagrams convert the odds to probabilities in the axes for the convenience of the user.

The use of the two likelihood ratios may be explained by an example. Suppose we have a test that has a specificity of 80% and sensitivity of 90%. Thus, the LR(+) = 4.5 and the LR(-) = 0.125. Suppose we have a prevalence of disease (pre-test probability) of 2%. Suppose further that we have a positive result from a patient without any known risk factors. The odds corresponding to the probability for having the disease is 0.02 / (1 – 0.02) = 0.020. Thus, the post-test odds of a positive result is 0.020 × 4.5 = 0.091, corresponding to a probability 8.4%. Suppose that the patient belongs to a sample population with a pre-test probability of 25% and we would like to know the probability that a negative value was true or false. The pre-test odds is 0.25 / (1 - 0.25) = 0.33, the post-test odds 0.33 x 0.125 = 0.041, corresponding to a probability of a true result of about 3%.

## ROC curve

The present report addresses diagnostic situations where increased (high) values are characteristic for a disease. In the opposite case, decreased values in disease, many of the variables will be mirrored.

The ROC curve, or the ROC space, is represented by a two dimensional diagram with the sensitivity (true positive rate) along the Y-axis and (1 – specificity) (false positive rate) along the X-axis. The categories can be anything that can be recognized and separated or classified by a quantity value, *e.g.* healthy and not healthy individuals.

Each point represents the sensitivity and (1 - specificity) for a particular cut-off value, *i.e.* modifying the relative number of those classified as true and false in the categories. Obviously, the coordinates of the points are equal to the LR(+) of the quantity value and correspond to the slope of the ROC curve (tangent) in that particular point. This is an alternative way to understand the relation between the post-test and the pre-test probability, *i.e.* the likelihood ratio.

**Figure 4:**
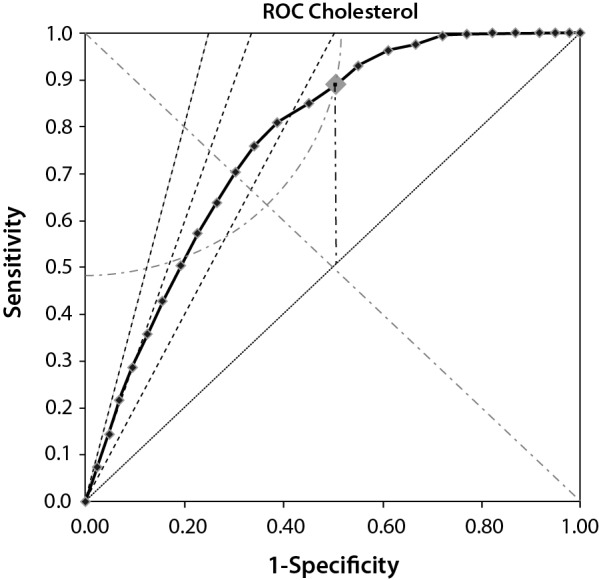
Indices and help-lines in a ROC diagram. The solid line is the ROC curve with the tested cut-off values indicated. Only points above the equality line (dotted line, Sensitivity = 1 - Specificity) have diagnostic power. The vertical line (dashed-dotted line) is the J-index calculated at the cut-off. The quarter-circle is the K-index at the cut-off and the oblique dashed lines are, from left to right, LR(+) = 4, 3 and 2, respectively.

The general pattern of the ROC curve shows a convex arc “standing” on the diagonal sensitivity = (1 – specificity), *i.e.* the “equality line” in the diagram (Figure 4); characterized by a LR(+) value of 1. The larger the LR(+) is, the larger is the gain by the test. ROC-curves that coincide with the equality line represent no gain at all and are regarded as a random guess.

The maximal outcome is when both the sensitivity and specificity is 1, this will correspond to a point in the upper left corner and be as far away from the equality line as possible.

The equality line summarizes all possible cut-offs which would generate a LR(+) of one. Therefore all points above the equality line indicate that the post-test probability will be higher that the pre-test probability and therefore of diagnostic interest. However we normally require a LR(+) above three to be really diagnostically useful. The lines corresponding to LR(+) equal to 2, 3 and 4 are shown in the graph (oblique dashed lines). The rational is that only outcomes above or to the left of these lines are really diagnostically useful. As shown in Figure 4 the available space with a LR(+) > 4 is reduced to a triangle with the base (1 - specificity = 0.25 at the sensitivity = 1) or 25% of the available “diagnostic” space.

Several indices are available to summarize the information in a ROC graph. The most established is the Youden index (J), which was explained mathematically already in the original publication (*5*-*7*). It is defined as J = sensitivity + specificity – 1.

The Youden index can take any value between - 1 (sensitivity or specificity = 0) and + 1 (sensitivity = specificity) which both represent extremes that are unlikely to occur in reality. The meaning of the J-index can be visualized in the ROC-plot. In Figure 4, a Youden index is indicated as a vertical dashed line from an arbitrarily chosen cut-off with the coordinates (1 - specificity_0_) / sensitivity_0_. By definition the X- and Y-coordinates are identical at the intersection of a vertical line with the equality line, *i.e.* (1 - specificity_1_) = sensitivity_1_. Therefore, the graphical representation of the J-index corresponds to sensitivity_0_ - (1 - specificity_1_) = sensitivity_0_ – sensitivity_1_. Clearly, the J-index is the distance between the ROC-curve and the equality line, an index of the efficiency of a cut-off. A maximal J-index will be the optimal choice of cut-off – provided it could be assumed that the sensitivity and specificity were diagnostically of equal importance. Other considerations may prevail.

Another useful index recently introduced is the K-index, which represents the distance from the upper left corner of the plot to the chosen cut-off representation on the ROC-curve (*8*, *9*). Since this is calculated using the Pythagoras theorem its graphical representation is a circle, a quarter of which is inside the ROC plot and shown in Figure 4. The interpretation is that any point inside the quarter-circle will perform better than those where the circle crosses the ROC curve. Note that the K-index crosses the ROC curve twice. This may have implications if the K-value is used to identify optimal cut-off values.

In case of symmetrical ROC-graph the J- and the K-index will coincide, with their interception lying on the negative diagonal, sensitivity = (1 - specificity).

## Cumulative data analysis

**Figure 5:**
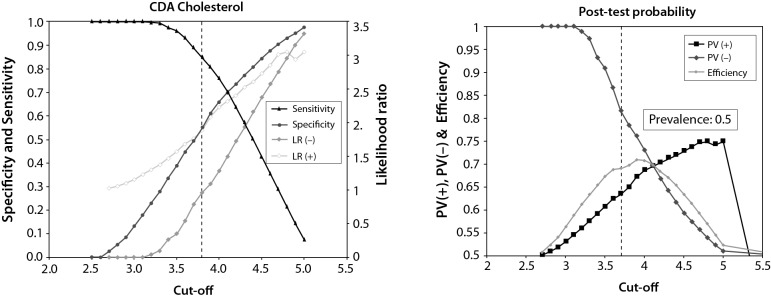
Cumulative data analysis (CDA) representation. The left panel shows the cumulative data plot. The cut-off has been transferred from the ROC-graph (Figure 4). The likelihood ratios (LR(+) and LR(-)), sensitivity and specificity are displayed. The right panel shows the probability plot. The actual values of the post-test probabilities are shown. PV (+) – positive predictive value. PV (-) – negative predictive value.

Although the ROC curve conveys a substantial amount of information, some is lost in the process, most importantly the value of the cut-off at each LR(+). Since it is not necessarily the maximum J-index that serves the clinic best, it may be important to offer a tool to infer the effect of modifying the cut-off value on the sensitivity, specificity and likelihood ratios. Therefore, an accompanying graph that relates the cut-off to these indicators is valuable. It summarizes the distributions and a suitable name is therefore cumulative data analysis (CDA) graph (*10*, *11*). In a spreadsheet application it linked to the ROC-graph. This allows graphical real-time effects of changing the cut-off value (Figure 5, left panel).

If a ‘rule-in’ is desirable then the specificity should be favoured, *i.e.* the cut-off moved to a higher value if this is linked to disease, in accordance with the model shown in Figure 1. Cut-off values which would generate a LR(+) in the optimal area (see Figure 4) are also displayed in the CDA plot. The example in Figure 5 (for cholesterol) indicates that a cut-off at 3.7 mmol/L would correspond to a LR(+) of 1.9. A higher LR(+) might be desirable and the highest we can get seems to be about 3 which would increase the specificity to about 0.9, whereas the sensitivity drops to 0.2 at a cut-off of 4.8 mmol/L. This cut-off, whether a reference value or decision value, would surely “rule in” individuals in the hypercholesterolemia group.

Given the prevalence of disease (pre-test probability) a graph of the post-test probabilities (predictive values of a negative of positive outcome, PV(-) and PV(+), respectively) encountered can be drawn (Figure 5, right panel) to illustrate the result of a change of the cut-off in a population with a given prevalence. In this particular example the prevalence was set to 50% for illustration purposes; if it is lower, the curves will be shifted to the right and eventually the chosen cut-off has no effect on the PV(+).

## Area under the curve

The area under the ROC curve (AUC) is the discriminatory ability of the test, *i.e.* ability to correctly classify the diseased and the healthy. It is also described as the probability that the test will rank a randomly chosen positive result higher than a randomly chosen negative result. Consequently, it is interesting to compare tests by comparing their AUC. Since the ROC curve is a summary of the LR(+) for every possible cut-off, trapezoids (a rectangular column with a triangle on top) can be visualized and their individual areas calculated as the average of two adjacent values of the sensitivity times the difference between the limiting (1- specificity) values. Their sum is an approximation of the AUC which obviously comes closer to the true value the more frequently the LR(+) is sampled, *i.e.* narrow columns, more cut-offs.

It was shown by Hanley and McNeil in a seminal publication in 1982 that the AUC is equivalent to the Wilcoxon U-statistics (*12*). This is a standard procedure to compare the medians of non-normal distributions, available in most statistics packages and easily calculated in a spreadsheet program *e.g.* Excel. The mechanics of the Wilcoxon signed rank test is to rank all observations and then add the rank of those which belong to the diseased and the non-diseased separately. If the sums are equal there is no difference between the groups which in the ROC translates to the equality line (sensitivity / (1 - specificity) = 1). In most programs the smallest U-value is evaluated; this, however, is not usually the desirable value in calculating the AUC and the U-value for the largest sum of ranks (usually the diseased) must be calculated. The U-value can be calculated as follows
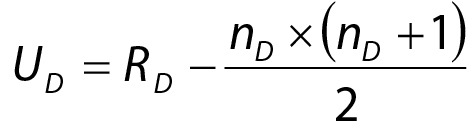
and subsequently the AUC is calculated as
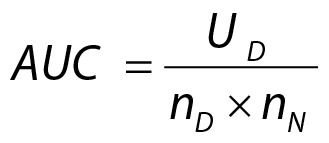
where R_D_ is the sum of ranks in the group with the largest sum of ranks, and n_D_ and n_N_ the number of observations in the groups.

## Comparison of AUCs

A common use of the AUC is to compare the performance of different methods. There are an unlimited number of ROC-curves with identical AUC and therefore the visual inspection of a ROC plot is essential. Often a visual inspection is sufficient to estimate the advantage of one of several methods but the AUC and its standard error of the mean can be calculated and the areas thus evaluated by the Student’s t-test.

An estimation of the standard error of the AUC was advised by Greiner *et al.* and described for a simplified model as:
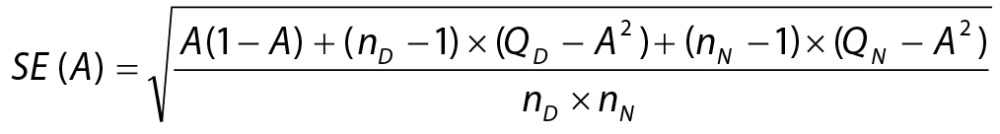


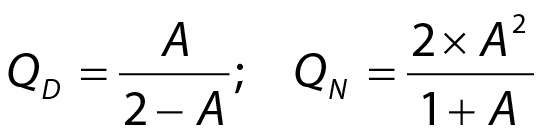
where SE(A) is the AUC’s standard error, n_D_ and n_N_ are the numbers of diseased and healthy examinees, respectively, and A is the AUC (*11*, *13*).

Access to the AUCs of two ROC and their standard errors allows estimating the significance of a difference between AUCs referring to a Students t-test:


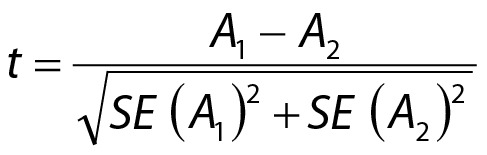


## Discussion

The contingency table can be organized differently with regard to which quantities will populate the various cells, but for many the interpretation seems to be based on a visual memory of the table. Since a readership concerned with medical diagnosis and comparisons of measurement procedures are familiar with scatter plots where high values of the independent variable (X-axis) correspond to high values of the dependent variable (Y-axis), it seems logical to copy this structure. Therefore, the number of diseased is in the first row, the number of negative results in the first column, accordingly, the true positives and the true negatives are placed along the positive diagonal of the table. This may be reversed if diseased are characterized by low values. Other designs are used in some literature.

Decisions are based on formulating, explicitly or implicitly, hypotheses and evaluating them in terms of probabilities. As soon as a physician faces a patient, a hypothesis on the reasons for complaints, based on a narrative and visual signs and symptoms will be (sub)consciously created. For an experienced physician this information may be sufficient for a diagnosis and subsequent treatment but modern techniques offer a wide variety of tests, which will narrow down the number of possible diagnoses. Rarely, however, will the outcome of a single test be unambiguous but if the test is carefully chosen the post-test probability for a correct hypothesis will be superior to the pre-test probability. It therefore becomes important for the physician to have a structured knowledge of the performance of requested tests. The Bayes’ theorem shows that the reliability of a test may depend on the prevalence of the disease; therefore, sequential application of tests may be beneficial even if ordering a wide selection of tests and investigations may be rational and practical in real life. Indeed, one of the most important skills of the clinician is to increase the pre-test probability before adding more tests.

The farther away from a cut-off value the less risk for false results, but false results become a major problem close to the reference interval limits. We need a tool to evaluate the risk for false answers; the false negatives (error Type II), which incorrectly dismiss a hypothesis, and false positives (error Type I) which falsely lead to unnecessary therapy. The sensitivity and specificity of the test are the key pieces of information and should be optimized considering the severity of the disease, the possibilities to treat and social consequences (*1*). These quantities are determined by the cut-off, or reference value, which separates diseased from non-diseased.

By definition, reference intervals cover the values found in a certain part, usually 95%, of a reference cohort and are defined to fit the purpose (*14*-*16*). The choice of reference interval does not consider or evaluate the risk for false results in relation to the measured quantity value. The risk within the reference interval is principally the same – a uniform of rectangular distribution. A reference cut-off may be defined differently, *e.g.* to include all diseased with a given probability or partitioned according to problem formulation or suspicion of a particular disease. Knowledge of the LR(+) values for different cut-offs would allow the physician to make decisions based on probabilities at certain quantity concentrations. This would serve “precision medicine” better than the rigid, population based, reference intervals. Presently much of the information in the laboratory results is lost because it is dichotomized. Probability based reference values would have a potency to recover lost ground.

Necessary information is not yet available but once accumulated it could easily be made available in hospital information systems or mobile nets *e.g.* smartphones. As Henderson and Rayana pointed out in 1985, this prompts us to report performance data in a complete and standardized format (*17*).

## Conclusion

Decisions are formally made according to Bayes’ theorem and their efficiency can be estimated, *i.e.* the effect on the performance by changing the cut-off values can be foreseen. The ROC plot is an excellent summary of the performance of a test but requires access to adequate computer support. Several of the indices, which may be difficult to communicate, can be included in the classical ROC plot. However, a ROC will not directly give the relation between the cut-off and the performance and therefore the CDA plot and visualization of the post-test probabilities are important complements and support in illustrating and deciding on the characteristics of a test. Risk assessment of results would be favoured by a redefinition of reference values and a personalized, dynamic use. Access to modern software *e.g.* spreadsheet programs makes establishing ROC and CDA plots possible for the individual laboratory and eventually made feasible by modern communication techniques.

A software package, coded in EXCEL, of the procedures described in the report is available from the author.

## Supplementary material

Appendix 1. Calculated quantities and their definitions

## References

[1] Galen RS, Gambino SR, editors. Beyond normality: Predictive value and efficiency of medical diagnoses. London: Churchill Livingstone, 1976.

[2] Henderson AR Assessing test accuracy and its clinical consequence: a primer for receiver operating characteristic curve analysis. Ann Clin Biochem. 1993;30:521–39. 10.1177/000456329303000601 8304720

[3] Zweig MH Campbell G Receiver-operating characteristics (ROC) plots. A fundamental evaluation tool in clinical medicine. Clin Chem. 1993;39:561–77. 8472349

[4] Ferard G, Dybkaer R, Fuentes-Arderiu X. IFCC-IUPAC: Compendium of terminology and nomenclature of properties in laboratory sciences. Recommendations 2016. London: The Royal Society of Chemistry, 2017.

[5] Fagan TJ Nomogram for Bayes’ Theorem. N Engl J Med. 1975;293:257. 10.1056/NEJM197507312930513 1143310

[6] Youden WJ Index for rating diagnostic tests. Cancer. 1950;3:32–5. 10.1002/1097-0142(1950)3:1<32::AID-CNCR2820030106>3.0.CO;2-3 15405679

[7] Fluss R Faraggi D Reiser B Estimation of the Youden index and its associated cutoff point. Biom J. 2005;47:458–72. 10.1002/bimj.200410135 16161804

[8] Kallner A, editor. Laboratory statistics. Waltham: Elsevier, 2017.

[9] Clinical and Laboratory Standards Institute (CLSI). Assessment of the diagnostic accuracy of laboratory tests using receiver operating characteristic curves; Approved guideline - Second Edition. CLSI document EP24-A2. Wayne (PA): CLSI, 2011.

[10] Bluestein BI Luderer AA Hess D Smith D Meyer KK Boyle G Measurement of ferritin-bearing peripheral mononuclear blood cells in cancer patients by radioimmunoassay. Cancer Res. 1984;44:4131–6. 10.1016/0022-1759(95)00121-P 6744324

[11] Greiner M Sohr D Göbel P A modified ROC analysis for the selection of cut-off values and the definition of intermediate results of serodiagnostic tests. J Immunol Methods. 1995;185:123–32. 10.1016/0022-1759(95)00121-P 7665894

[12] Hanley JA McNeil BJ The meaning and use of the area under the receiver operating characteristic (ROC) Curve. Radiology. 1982;143:29–36. 10.1148/radiology.143.1.7063747 7063747

[13] Beck JR Shultz EK The use of relative operating characteristic (ROC) curves in test performance evaluation. Arch Pathol Lab Med. 1986;110:13–20. 3753562

[14] Gräsbeck R Fellman J Normal values and statistics. Scand J Clin Lab Invest. 1968;21:193–5. 10.3109/00365516809076984 5708691

[15] Gräsbeck R Saris NE Establishment and use of normal values. Scand J Clin Lab Invest. 1969; Suppl 110:62–3.

[16] Solberg HE International Federation of Clinical Chemistry (IFCC), Scientific committee, Clinical Section, Expert panel on theory of reference values, and International Committee for Standardization in Haematology (ICSH), Standing Committee on Reference Values. Approved recommendation (1986) on the theory of reference values. Part 1. The concept of reference values. J Clin Chem Clin Biochem. 1987;25:337–42. 3612033

[17] Henderson AR Bhayana V A modest proposal for the consistent presentation of ROC plots in clinical chemistry. Clin Chem. 1995;41:1205–6. 7628106

