# Supplementary figures and images for: Bayes’ theorem, the ROC diagram and reference values: Definition and use in clinical diagnosis
Source: Biochem Med (Zagreb). 2017 Nov 24;28(1):010101. doi: 10.11613/BM.2018.010101 (PMC5707117; doi:10.11613/BM.2018.010101)

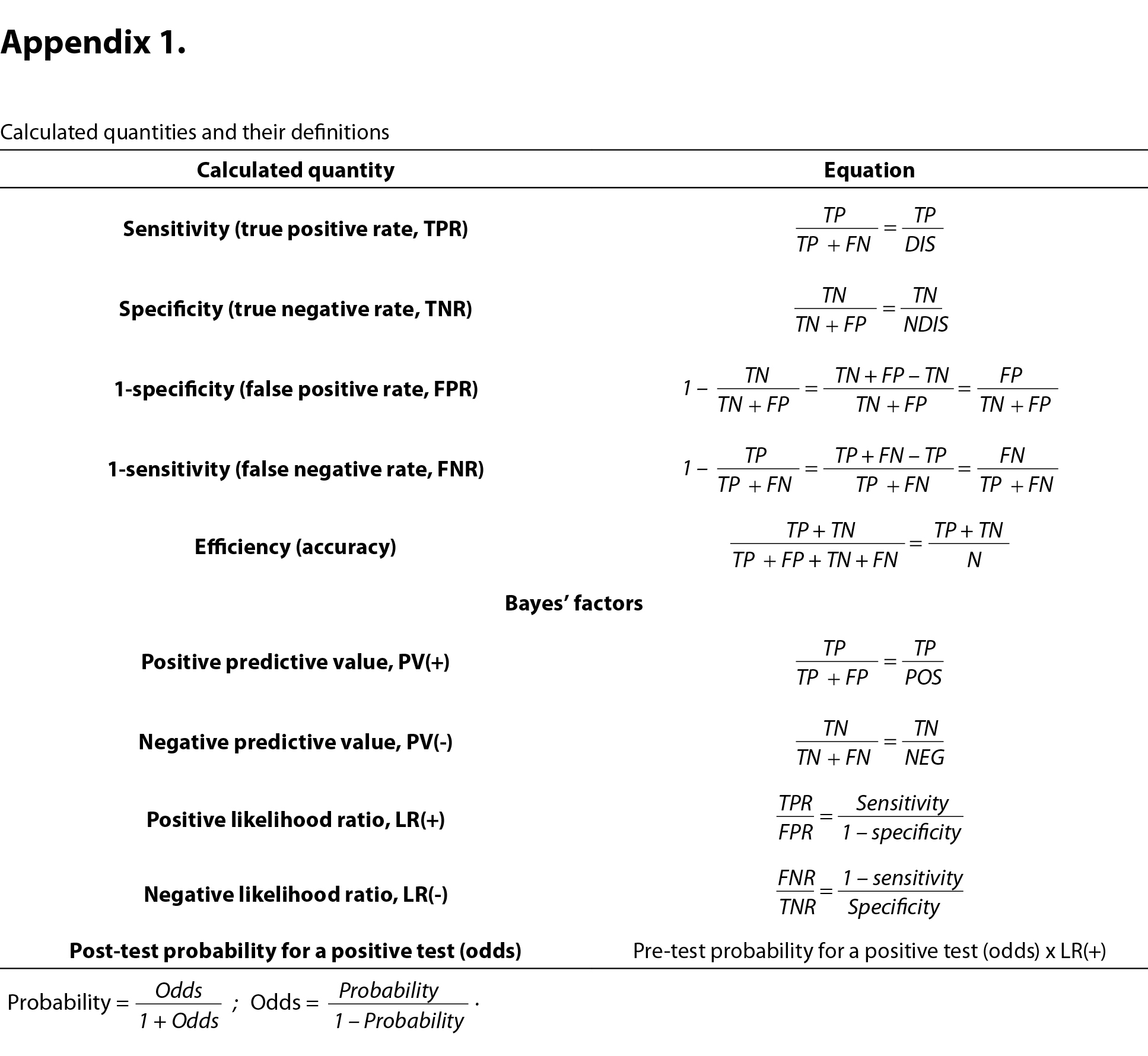

Supplement: Supplementary file 1 — Appendix 1. Calculated quantities and their definitions [file bm-28-1-010101-S1.tif]
